# Synthesis of multi-lactose-appended β-cyclodextrin and its cholesterol-lowering effects in Niemann–Pick type C disease-like HepG2 cells

**DOI:** 10.3762/bjoc.13.2

**Published:** 2017-01-03

**Authors:** Keiichi Motoyama, Rena Nishiyama, Yuki Maeda, Taishi Higashi, Yoichi Ishitsuka, Yuki Kondo, Tetsumi Irie, Takumi Era, Hidetoshi Arima

**Affiliations:** 1 Graduate School of Pharmaceutical Sciences, Kumamoto University, 5-1 Oe-honmachi, Chuo-ku, Kumamoto 862-0973, Japan; 2 Program for Leading Graduate Schools “HIGO (Health life science: Interdisciplinary and Glocal Oriented) Program”, Kumamoto University, 5-1 Oe-honmachi, Chuo-ku, Kumamoto 862-0973, Japan; 3 Department of Cell Modulation, Institute of Molecular Embryology and Genetics, Kumamoto University, 2-2-1 Honjo, Chuo-ku, Kumamoto 860-0811, Japan

**Keywords:** asialoglycoprotein receptor, cholesterol, cyclodextrin, lactose, Niemann–Pick type C disease

## Abstract

Niemann–Pick type C (NPC) disease, characterized by intracellular accumulation of unesterified cholesterol and other lipids owing to defects in two proteins NPC1 and NPC2, causes neurodegeneration and other fatal neurovisceral symptoms. Currently, treatment of NPC involves the use of 2-hydroxypropyl-β-cyclodextrin (HP-β-CD). HP-β-CD is effective in the treatment of hepatosplenomegaly in NPC disease, albeit at a very high dose. One of the methods to reduce the required dose of HP-β-CD for treatment of NPC is to actively targeting hepatocytes with β-cyclodextrin (β-CD). The aim of the present study was to synthesize a novel multi-lactose-appended β-CD (multi-Lac-β-CD) and to evaluate its cholesterol-lowering effect in U18666A-treated HepG2 (NPC-like HepG2) cells. Further, the study aimed at delivering β-CD to hepatocytes via cholesterol-accumulated HepG2 cells, and indicated that the newly synthesized multi-Lac-β-CD had an average degree of substitution of lactose (DSL) of 5.6. This newly synthesized multi-Lac-β-CD was found to significantly decrease the concentration of intracellular cholesterol with negligible cytotoxicity as compared to HP-β-CD. An increased internalization of TRITC-multi-Lac-β-CD (DSL 5.6) as compared to TRITC-HP-β-CD was observed in NPC-like HepG2 cells. Further, the dissociation constant of peanut lectin with multi-Lac-β-CD (DSL5.6) was found to be extremely low (2.5 × 10^−8^ M). These results indicate that multi-Lac-β-CD (DSL5.6) diminished intracellular cholesterol levels in NPC-like HepG2 cells via asialoglycoprotein receptor (ASGPR)-mediated endocytosis.

## Introduction

The Niemann–Pick type C (NPC) disease is a lipid storage disorder with the accumulation of membrane lipids such as cholesterol and multiple sphingolipids especially in lysosomes. The excessive accumulation of these lipids results in a progressive neurologic and visceral dysfunction [1–3]. A mutation in the *NPC1* and/or *NPC2* gene causes the NPC disease. The majority of the mutations occur in the *NPC1* gene that produces NPC1 protein responsible for binding [3] and transporting of cholesterol to the endolysosomes [4–5]. Meanwhile the remaining mutations are located in the *NPC2* gene that encodes the NPC2 protein as a lysosomal protein. Excessive accumulation of cholesterol in endolysosomes in children, results in severe hepatosplenomegaly and neurodegeneration [3,6]. Hence, the sequestration of cholesterol is an important factor in the development of the NPC disease.

Cyclodextrins (CDs) are cyclic glucose oligosaccharides used by the pharmaceutical industry to enhance solubility, stability, and bioavailability of drugs. CDs improve the above properties of drugs through complex formation, when formulated as injectable solutions, sprays, eye drops, powders, and tablets [7–8]. In recent years, the efficacy of 2-hydroxypropyl-β-cyclodextrin (HP-β-CD) in the treatment of the NPC disease has attracted considerable attention [9–10]. Administration of HP-β-CD to *NPC1*-knockout (*Npc1^−/−^*) mice significantly prolonged the survival rate by reducing the cholesterol levels [9–11]. In addition, therapeutic effects of HP-β-CD have been observed in mouse models of NPC disease. These studies have led to the initiation of two human clinical trials, a phase I/IIa trial in 2013 and a phase IIb/III trial in 2015, by the National Institutes of Health (NIH), USA. However, the administration of high doses of HP-β-CD is inevitable to obtain the effects in vivo because HP-β-CD cannot enter the cells owing to its hydrophilicity and relatively high molecular weight.

The asialoglycoprotein receptor (ASGPR), a hepatic galactose or *N*-acetylglucosamine (GlcNAc) receptor, is highly expressed on the sinusoidal cell surface of hepatocytes. ASGPR is responsible for the binding, internalization, and subsequent clearance of glycoproteins containing terminal galactose or GlcNAc residues from the circulation [12]. Hence, galactosylated nanocarriers have been utilized for the selective delivery of drugs to the liver via ASGPR-mediated endocytosis [13]. In fact, ASGPR-mediated endocytosis is one of the promising approaches for delivery of CDs into hepatocytes for the treatment of hepatosplenomegaly in NPC disease. In our previous report, monolactosylated β-CD (mono-Lac-β-CD) diminished the cholesterol accumulation in U18666A-treated HepG2 cells (NPC-like HepG2 cells), as a model of NPC hepatocytes, as much as HP-β-CD [14]. Further, ASGPR recognizes galactose moieties at three points, collectively known as the “golden triangle” [15–16]. However, as mono-Lac-β-CD can bind to only one point in the golden triangle of ASGPR, it has insufficient binding ability to ASGPR.

Consequently, in the present study, we synthesized a novel multi-lactose-appended β-CD (multi-Lac-β-CD) to enhance the targeting ability to ASGPR, and evaluated its cholesterol-lowering effect in NPC-like HepG2 cells.

## Results and Discussion

### Synthesis of multi-Lac-β-CD

**Figure 1:**
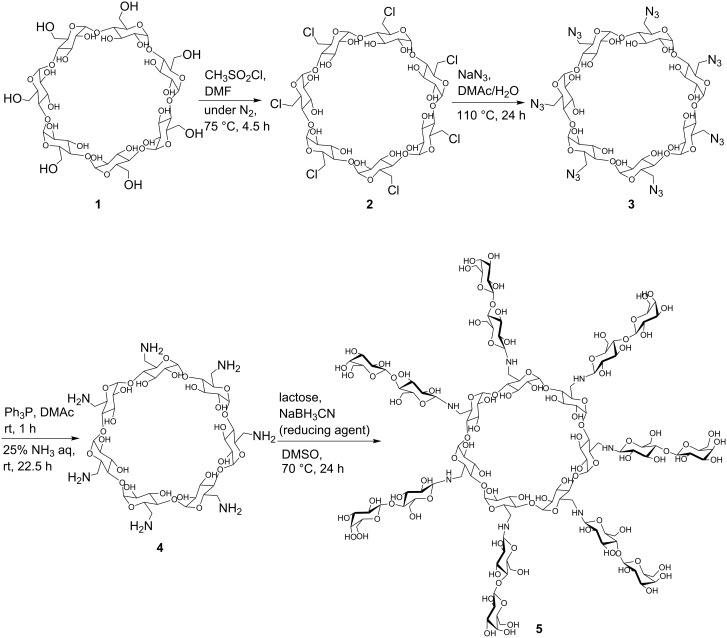
Schematic representation for synthesis of multi-Lac-β-CD (**5**), β-CD (**1**), *per*-chloro-β-CD (**2**), *per*-azido-β-CD (**3**), *per*-amino-β-CD, (**4**), multi-Lac-β-CD (DSL5.6), DSL, degree of substitution of lactose, DMAc, dimethylacetamide.

**Figure 2:**
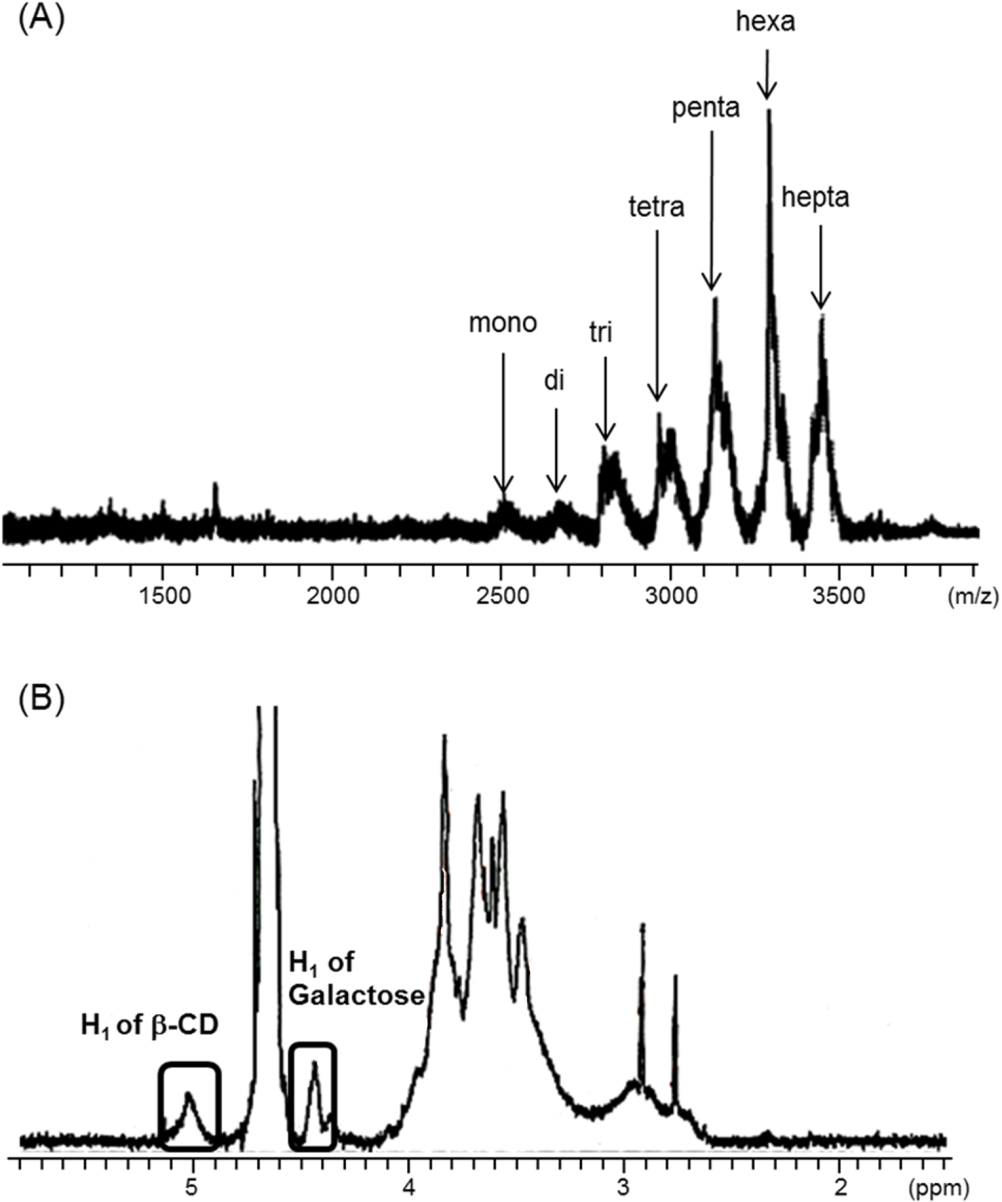
MALDI–TOFMS (A) and ^1^H NMR spectra (B) of multi-Lac-β-CD (DSL5.6)

In the present study, we fabricated multi-Lac-β-CD (**5**) to obtain a hepatocyte-specific cholesterol-lowering agent. The schematic representation of the synthesis of multi-Lac-β-CD (**5**) is shown in Figure 1. Briefly, *per*-NH_2_-β-CD (**4**) was synthesized by chlorination (*per*-chloro-β-CD (**2**)) and azidation (*per*-azido-β-CD (**3**)) of primary hydroxy groups of β-CD **1**, as reported previously [17]. It may be possible to use *per*-iodo- or *per*-bromo-β-CD instead of *per*-chloro-β-CD. However, the iodo-β-CD may be unstable due to the too high reactivity. Therefore, we prepared *per*-chloro-β-CD with sufficient reactivity. Finally, lactose groups were inserted in *per*-amino-β-CD by treatment with cyanotrihydroborate in dimethyl sulfoxide (DMSO) at 90 °C for 24 h. The product yield of multi-Lac-β-CD was 25%, and no unreacted compounds were confirmed by thin-layer chromatography (TLC). The MALDI–TOF MS spectrum of multi-Lac-β-CD revealed several peaks at *m*/*z* 2804, *m*/*z* 3006, *m*/*z* 3139, *m*/*z* 3296*,* and *m*/*z* 3454, corresponding to tri-, tetra-, penta-, hexa- and hepta-lactose-substituted β-CDs, respectively (Figure 2A). Further, the ^1^H NMR spectrum indicated that the degree of substitution of lactose (DSL) was 5.6. This value was obtained from the integral values of the anomeric protons of lactose and glucose in β-CD (Figure 2B). These results indicate the successful synthesis of multi-Lac-β-CD (DSL5.6).

### Cytotoxicity of multi-Lac-β-CD (DSL5.6)

**Figure 3:**
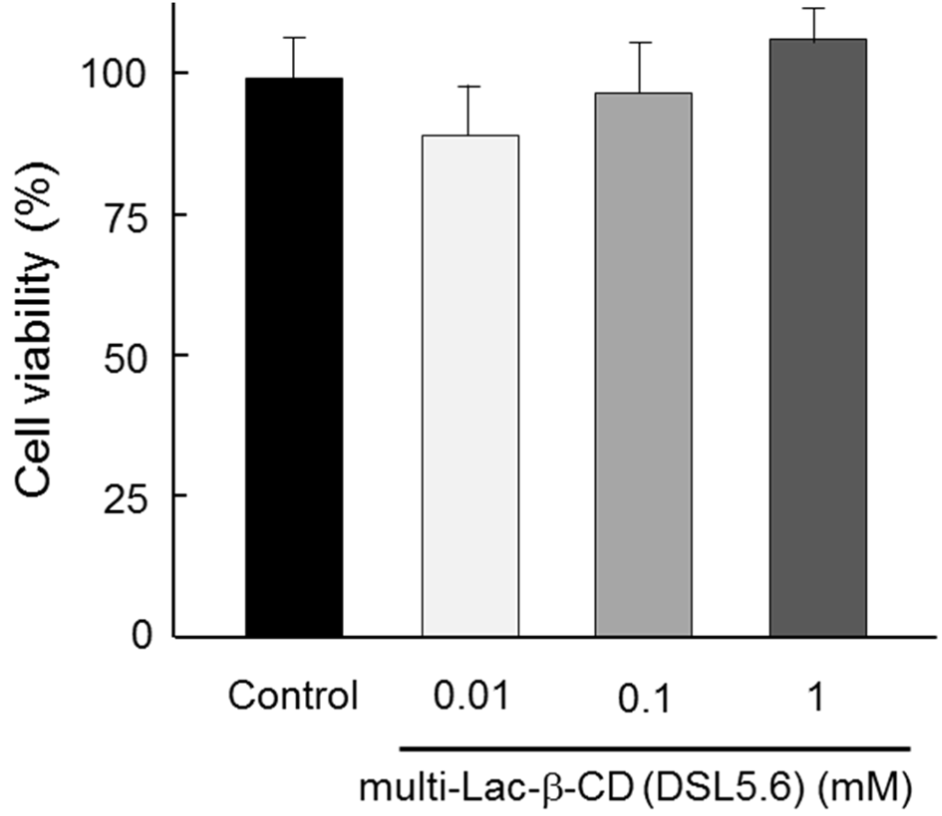
Cytotoxicity of multi-Lac-β-CD in NPC-like HepG2 cells after treatment for 24 h. NPC-like HepG2 cells were incubated with 150 μL of media [FBS (–)] containing multi-Lac-β-CD (DSL5.6) at 37 °C for 24 h. Data represents mean ± S.E.M. for six experiments.

To evaluate the cytotoxicity of multi-Lac-β-CD (DSL5.6), cell viability was examined after treatment with multi-Lac-β-CD (DSL5.6) by the water-soluble tetrazolium (WST)-1 method (Figure 3). U18666A-treated HepG2 cells were used as NPC-like cells in this experiment because U18666A can inhibit cholesterol trafficking in cells and simulate NPC disease phenotypes [18]. No significant changes in cytotoxicity were observed in NPC-like HepG2 cells after treatment with 0.01–1 mM multi-Lac-β-CD (DSL5.6) for 24 h.

### Interaction between multi-Lac-β-CD (DSL5.6) and peanut agglutinin

**Figure 4:**
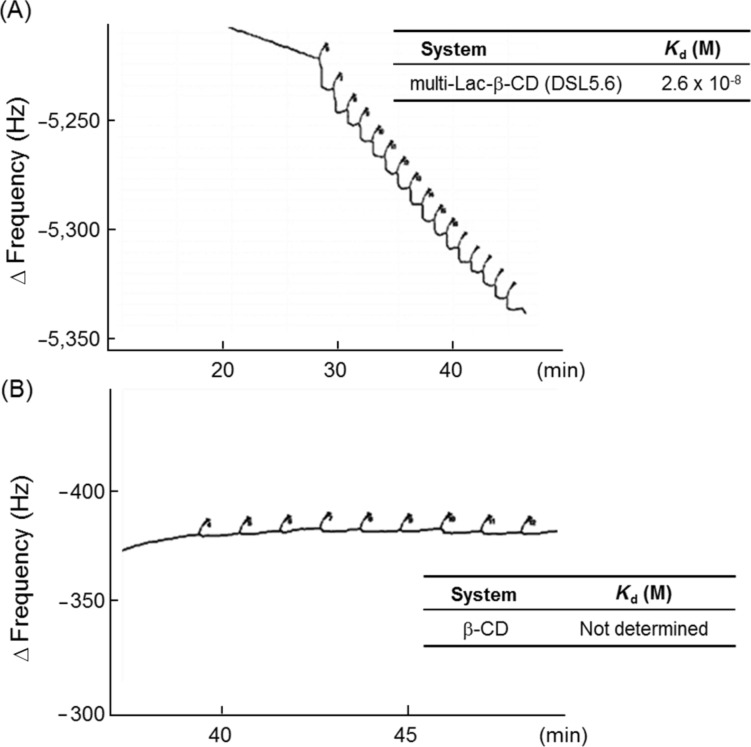
Binding curves of multi-Lac-β-CD (DSL5.6) (A) and β-CD (B) with peanut agglutinin (PNA). The binding curves for multi-Lac-β-CD (DSL5.6) and β-CD were determined by the quartz crystal microbalance (QCM) method. Phosphate-buffered saline (pH 7.4) was used as the sample buffer.

We initially attempted to clarify the role of the lactose moieties in multi-Lac-β-CD for binding to ASGPR by determining the dissociation constant of multi-Lac-β-CD (DSL5.6) with peanut agglutinin (PNA), a galactose-binding lectin, using the quartz crystal microbalance method (QCM) (Figure 4A). The dissociation constant of multi-Lac-β-CD (DSL5.6) was found to be 2.6 × 10^−8^ M, which was comparable with that obtained for the positive control asialofetuin (AF; 3 × 10^−8^ M). In contrast, β-CD did not show any response in the frequency of detection unit of QCM, thus indicating a negligible interaction with PNA (Figure 4B). Hence, the data indicated specific galactose-binding ability of multi-Lac-β-CD (DSL5.6).

### Intracellular distribution of TRITC-multi-Lac-β-CD (DSL5.6)

**Figure 5:**
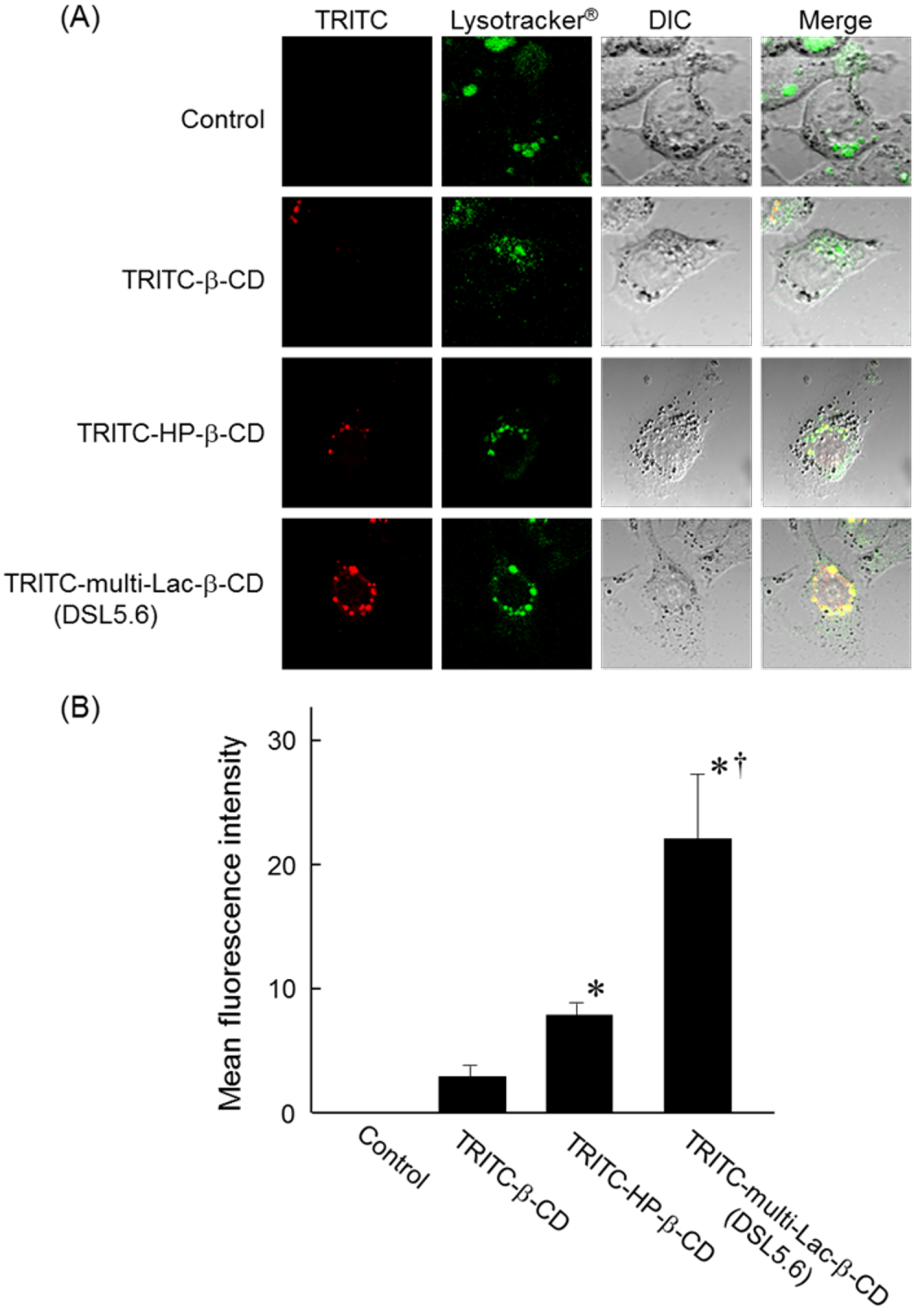
Intracellular distribution of TRITC-multi-Lac-β-CD (DSL5.6) in NPC-like HepG2 cells. Cells were incubated in medium [FBS (−)] with or without 100 μM TRITC-CDs for 24 h. (B) The fluorescence intensities of TRITC-CDs were determined in ten randomly selected cells per image by confocal laser scanning microscopy and BZ-II analyzer. Data represents mean ± S.E.M. of three experiments. *p < 0.05, compared with TRITC-β-CD. ^†^*p* < 0.05, compared with 100 μM TRITC-HP-β-CD.

Next, to investigate whether multi-Lac-β-CD (DSL5.6) can enter ASGPR-expressing cells, we examined the intracellular distribution of tetramethylrhodamine isothiocyanate (TRITC)-multi-Lac-β-CD (DSL5.6) in NPC-like HepG2 cells (ASGPR positive cells) Figure 5. The cellular uptake of TRITC-multi-Lac-β-CD (DSL5.6) was observed in NPC-like HepG2 cells 24 h post incubation (Figure 5A). Additionally, TRITC-multi-Lac-β-CD (DSL5.6) was co-localized with the endolysosomes stained by LysoTracker^®^. The fluorescence intensity of TRITC in cells was measured using the software of the BZ-II analyzer (Figure 5B). Meanwhile, the intracellular levels of TRITC-β-CD and TRITC-HP-β-CD in NPC-like HepG2 cells were observed to be lower than that of TRITC-multi-Lac-β-CD (DSL5.6) (Figure 5B). In summary, these results indicate that multi-Lac-β-CD (DSL5.6) was distributed to the endolysosomes after cellular uptake by the NPC-like HepG2 cells.

### Effects of multi-Lac-β-CD (DSL5.6) on intracellular cholesterol levels

**Figure 6:**
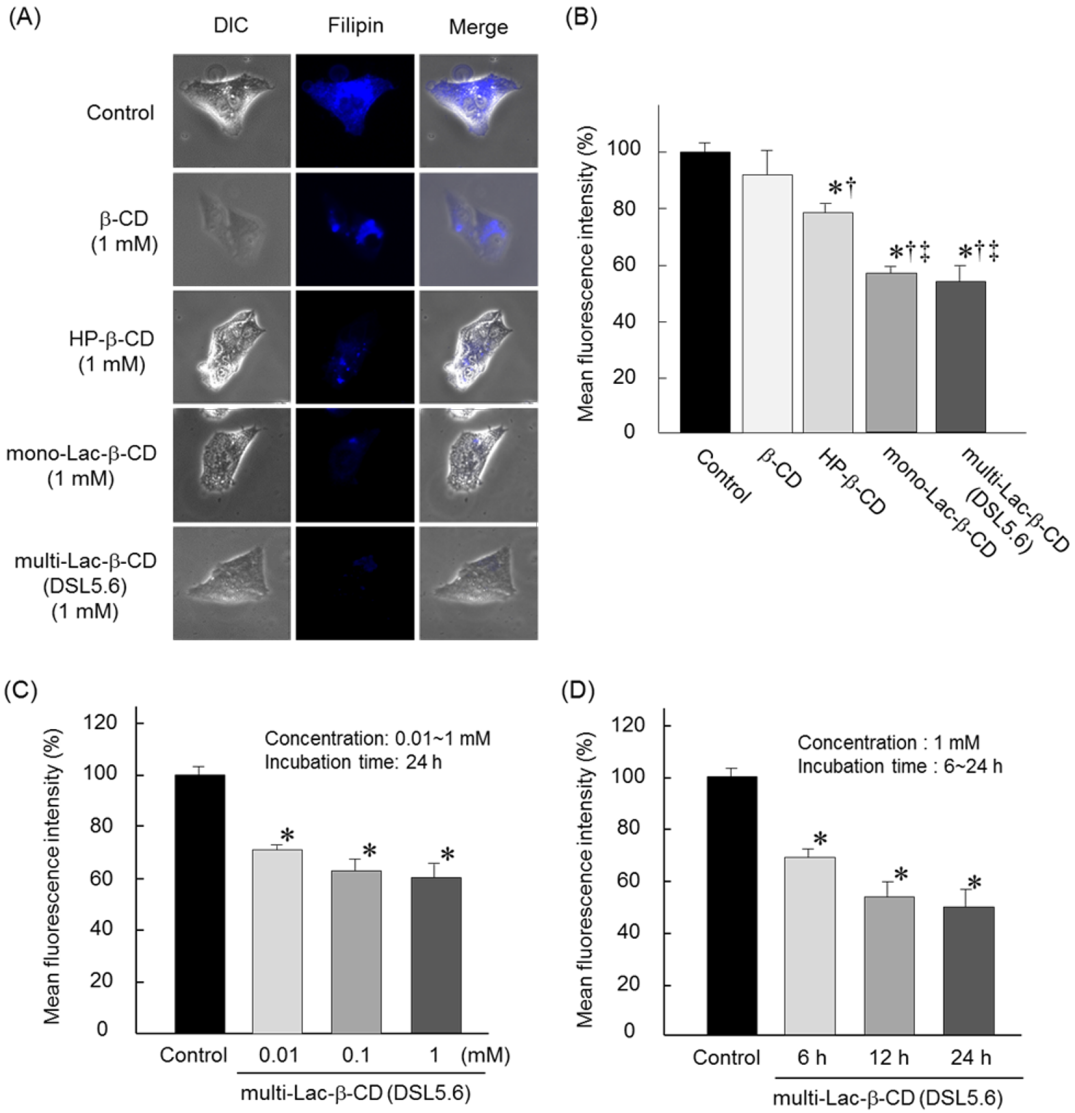
Intracellular distribution of cholesterol in NPC-like HepG2 cells. (A) NPC-like HepG2 cells (1 × 10^5^ cells/35 mm dish) were treated with β-CD, HP-β-CD, mono-Lac-β-CD or multi-Lac-β-CD (DSL5.6) for 24 h at a concentration of 1 mM. (B) Fluorescence intensities of Filipin III in ten randomly selected cells were determined by the fluorescence microscope and BZ-II analyzer. (C) Effects of varying concentrations of multi-Lac-β-CD (DSL5.6) on cholesterol levels. (D) Effects of incubation time of multi-Lac-β-CD (DSL5.6) on cholesterol levels. Data represent mean ± S.E.M. of three experiments. *p < 0.05, compared with control. ^†^*p* < 0.05, compared with β-CD. ^‡^*p* < 0.05, compared with HP-β-CD.

The effects of β-CD, HP-β-CD, mono-Lac-β-CD and multi-Lac-β-CD (DSL5.6) on intracellular cholesterol levels were studied in NPC-like HepG2 cells using Filipin III, which can specifically bind to unesterified cholesterol (Figure 6). Treatment of cells with 1 mM HP-β-CD, mono-Lac-β-CD and multi-Lac-β-CD (DSL5.6) for 24 h diminished the Filipin III-derived fluorescence intensity, when compared to that with β-CD (Figure 6A). These results indicated a significant cholesterol-lowering effect of multi-Lac-β-CD (DSL5.6) with statistical difference, as compared to that of β-CD or HP-β-CD (Figure 6B). In addition, the cholesterol-lowering effect of multi-Lac-β-CD (DSL5.6) tended to be stronger than that of mono-Lac-β-CD, although there was no significant difference between these Lac-β-CDs (Figure 6A and 6B). Furthermore, this cholesterol-lowering effect of multi-Lac-β-CD (DSL5.6) was concentration- and time-dependent (Figure 6C and 6D). Hence, these results indicated that multi-Lac-β-CD (DSL5.6) decreased intracellular cholesterol levels in HPC-like HepG2 cells.

HP-β-CD, one of the therapeutic candidates for the treatment of NPC disease, attenuates free cholesterol accumulation in various organs and prolongs the lifespan of *Npc1*^−/−^ mice and NPC-diseased human fibroblasts [9–10,19]. Toxicological studies indicate that HP-β-CD is generally safe. However, a recent study has found that repeated administration of high doses of HP-β-CD causes hearing loss in cats [20–21]. Therefore, the targeting technique is one of promising approaches to reduce the dose of CDs. Importantly, the multi-Lac-β-CD (DSL5.6) showed more potent cholesterol-lowering effects in NPC-like HepG2 cells than HP-β-CD. Accordingly, multi-Lac-β-CD (DSL5.6) may attenuate the risk of hearing loss due to its hepatocyte-selectivity. In order to evaluate the liver accumulation of multi-Lac-β-CD (DSL5.6), the biodistribution studies need to be performed in NPC disease model mice in future.

The galactose density is a crucial factor for regulation of galactose affinity to ASGPR. Stokmaier et al. revealed that the binding affinity of galactose to ASGPR elevated 100–1000 fold from mono- to triantennary galactose structures, probably due to clustering effects [22]. The dissociation constant of monosaccharide with ASGPR was 10^–4^ M, whereas those of triantennary and tetraantennary compounds with ASGPR were 5 × 10^−9^ M and 9 × 10^−9^ M, respectively [22]. Previously, we synthesized the mono-Lac-β-CD and revealed the reduction in accumulation of intracellular cholesterol in NPC-like HepG2 cells [14]. ASGPR is known to recognize the galactose moiety at three points in the golden triangle [15–16]. Therefore, the binding affinity of multi-Lac-β-CD (DSL5.6) to ASGPR is likely to be much higher than that of mono-Lac-β-CD. In fact, multi-Lac-β-CD (DSL5.6) has a quite low dissociation constant (2.6 × 10^−8^ M) with PNA, probably a result of its clustering effect in ASGPR recognition. The further elaborate studies to compare the binding affinity of multi-Lac-β-CD (DSL5.6) to PNA with mono-Lac-β-CD are necessary. In addition, to demonstrate the ASGPR-expressing cell-selective binding and cholesterol-lowering effects of multi-Lac-β-CD (DSL5.6), the comparative studies using ASGPR-negative cells are also needed.

Several lines of evidence indicate that the lowering effect of CDs on the accumulation of cholesterol is attributed to endocytosed CD [23]. The CDs can enter the cells via pinocytosis and reach the interior of the endolysosomes [24] and should remain intact because mammalian cells lack the enzymes for degradation of CDs [8]. A current study indicates that endocytosed CDs are capable of replacing the function of NPC1 [23]. However, it is still unclear how the endocytosed CDs can bypasses the functional NPC1. NPC1, a transmembrane protein expressed in endolysosomes, can bind to cholesterol [25]. NPC2 interacts with NPC1 and delivers cholesterol to the limiting membranes [25], while β-CDs form inclusion complexes with cholesterol. Hence, β-CDs may have the potential to bypass the functional NPC1 or NPC2 and deliver cholesterol directly to the limiting membranes. Once the cholesterol exits the endolysosomes, it is delivered to other organelles by the cholesterol transport system. To elucidate the detailed mechanism of cholesterol-lowering effects of multi-Lac-β-CD (DSL5.6), further elaborate studies on not only the interaction of multi-Lac-β-CD (DSL5.6) with endolysosomes membranes, but also cholesterol trafficking are necessary.

## Conclusion

In this study, we developed multi-Lac-β-CD (DSL5.6) to reduce cholesterol levels in NPC-like HepG2 cells. The multi-Lac-β-CD (DSL5.6) was internalized in NPC-like HepG2 cells via ASGPR-mediated endocytosis and was found to diminish the accumulation of cholesterol in the endolysosomes. In conclusion, the findings from the present study may be crucial for the development of an active targeting CD-based therapy for the treatment of hepatosplenomegaly in NPC disease.

## Experimental

### Materials

β-CD was a kind gift from Nihon Shokuhin Kako (Tokyo, Japan). Lactose monohydrate was purchased from Wako Pure Chemical Industries (Osaka, Japan). Tetramethylrhodamine isothiocyanate (TRITC) was obtained from Funakoshi (Tokyo, Japan). Biotinylated-peanut agglutinin (PNA) and bovine serum albumin (BSA) were purchased from Cosmo Bio (Tokyo, Japan) and Boehringer Mannheim K.K. (Tokyo, Japan), respectively.

### Synthesis of multi-Lac-β-CD (DSL5.6)

*per*-6-Amino-β-CD (**4**) was synthesized as reported previously [17]. Lactose residues were introduced in the primary amino groups of *per*-6-amino-β-CD. For this experiment, 20 mL of DMSO containing *per*-6-amino-β-CD (**4**, 0.1 g), lactose monohydrate (1.52 g), and sodium cyanotrihydroborate (2.79 g) were mixed and incubated at 70 °C for 24 h. After dialysis by Spectra/pore (MWCO: 1000) in water at room temperature for 48 h, the sample was concentrated in a rotary evaporator and freeze-dried to obtain multi-Lac-β-CD. ^1^H NMR spectra for multi-Lac-β-CD consisted of protons of both β-CD and lactose. The ratios of peak areas of the anomeric proton of β-CD and protons of the lactose were approximately 5.6. This result indicated that β-CD covalently binds to lactose in a molar ratio of 1:5.6 (Figure 2B). A 25% yield of multi-Lac-β-CD was obtained after synthesis. ^1^H NMR (500 MHz, D_2_O) δ (from TMS), 5.00 (H1, β-CD), 3.84−3.62 (H3, H5, H6, β-CD), 3.54−3.43 (H2, H4, β-CD), 4.56−4.41 (anomeric proton of lactose).

### Cell culture

HepG2 cells (human hepatocellular carcinoma cell line) were grown in Dulbecco's Modified Eagle's Medium (DMEM, Nissui Pharmaceuticals, Tokyo, Japan), containing penicillin (1 × 10^5^ mU/mL), streptomycin (0.1 mg/mL) and supplemented with 10% fetal bovine serum (FBS), in a humidified incubator with 5% CO_2_. NPC-like HepG2 cells accumulated with high amounts of cholesterol were obtained after 48 h treatment with DMEM containing 1.25 μM U18666A.

### Cytotoxicity

The viability of NPC-like HepG2 cells treated with multi-Lac-β-CD (DSL5.6) was assayed by the WST-1 method. A cell counting kit (CCK) from Wako Pure Chemical Industries (Osaka, Japan) was used for the analysis. The WST-1 method has been described in-depth in our previous publication [14].

### Quartz crystal microbalance (QCM)

The molecular interaction of multi-Lac-β-CD (DSL5.6) with PNA, one of galactose-binding lectins, was analyzed with a QCM analyzer (Single-Q, SCINICS, Tokyo, Japan). Avidin was used for immobilizing biotinylated-PNA on the sensor cuvette. The biotinylated-PNA was blocked with 1% BSA in PBS (pH 7.4). Two μL of multi-Lac-β-CD (DS5.6) (final concentration: 1 mM) was added to PBS (pH 7.4). The binding curves and dissociation constants (*K*_d_) were obtained using Q-UP software equipped in the Single-Q.

### Intracellular distribution of multi-Lac-β-CD (DSL5.6)

The intracellular distribution of TRITC-multi-Lac-β-CD (DS5.6) in NPC-like HepG2 cells was assayed by confocal laser scanning microscopy, as reported previously [14].

### Intracellular cholesterol levels post treatment with multi-Lac-β-CD (DSL5.6)

The intracellular cholesterol levels in multi-Lac-β-CD (DS5.6) treated NPC-like HepG2 cells were detected by a cholesterol cell-based detection assay kit (Cayman Chemical Company, Ann Arbor, MI, USA), as reported previously [14]. The fluorescence intensities of Filipin III in ten randomly selected cells were calculated as the ratio of Filipin III area to cell area by the fluorescence microscope and BZ-II analyzer.

### Data analysis

Quantitative data was expressed as the mean ± standard error of the mean (S.E.M.), while the statistical comparisons were made using the Scheffe's test. A *p*-value < 0.05 was considered statistically significant.
